# Improving information sharing in Medicaid home and community‐based services with learning health systems: Implications for older adults and individuals living with disabilities and dementia

**DOI:** 10.1002/lrh2.70029

**Published:** 2025-08-01

**Authors:** Chanee D. Fabius, Christin Diehl, Quincy M. Samus, Joseph J. Gallo, Jennifer L. Wolff

**Affiliations:** ^1^ Johns Hopkins Bloomberg School of Public Health Baltimore Maryland USA; ^2^ The Hilltop Institute University of Maryland Baltimore County Baltimore Maryland USA; ^3^ Johns Hopkins University School of Nursing Baltimore Maryland USA

**Keywords:** dementia, home care, long‐term services and supports, Medicaid home and community‐based services

## Abstract

**Introduction:**

Medicaid home and community‐based services (HCBS) support community living for older adults and individuals living with disabilities. Information sharing and effective communication among home care team members are critical to HCBS care coordination but are often fragmented, resulting in potentially avoidable consequences, particularly for persons living with complex health conditions, like dementia. Medicaid HCBS programs collect a range of data that could be leveraged in a learning health system (LHS) model to strengthen coordination between home care team members to improve outcomes and equity in HCBS care delivery.

**Methods:**

We leverage Friedman's Socio‐technical Infrastructure for LHS to consider information sharing capabilities and needs within Medicaid HCBS in Maryland.

**Results:**

Given longitudinal care complexities, significant costs, and data collection protocols, Medicaid HCBS is promising for LHS efforts. Recommendations for implementing an LHS in Medicaid HCBS highlight a socio‐technical infrastructure (i.e., people, technology, policies, processes), information cycles (e.g., existing research relationships and opportunities for expansion), and governance (e.g., ensuring ethical LHS implementation). Additional considerations for persons living with dementia should be made, such as the integration of dementia‐related policies into care delivery.

**Conclusions:**

Integrating LHS strategies into Medicaid HCBS holds promise for strengthening efficiency and equity in information sharing across state agencies, care teams (e.g., direct care workers, nurses), and families.

## 1 INTRODUCTION

Medicaid is the primary payer for long‐term services and supports (LTSS) in the U.S.^1^ There has been a profound transformation in the Medicaid LTSS landscape toward community living over the past several decades. In 1990, nearly 90% of Medicaid spending on LTSS was directed to institutional care,^2^, ^3^ whereas by 2020, 62% of Medicaid LTSS spending was directed to home and community‐based services (HCBS).^3^ Medicaid HCBS are designed to enable community living for people living with disabilities and low incomes.^4^, ^5^, ^6^, ^7^ States administer Medicaid HCBS to deliver services that support routine daily activities, such as homemaking, personal care, and home‐delivered meals. Due to varied levels of state financial support and programmatic commitment to HCBS (relative to mandatory institutional care) and complex eligibility policies, the national landscape of Medicaid HCBS is tremendously heterogeneous.^5^ Contributing to the variation from state to state, the delivery of Medicaid HCBS is often characterized by the complex and fragmented ways in which information is shared and services are coordinated.^8^

The rebalancing of state priorities from institutional to community‐based care has enabled individuals living with greater levels of disability to live in settings of their choice. In 2020, nearly 4.2 million Medicaid beneficiaries were using Medicaid HCBS.^1^ Medicaid HCBS participants consist of a range of individuals with high‐level needs, including intellectual and developmental disabilities, serious mental and behavioral health conditions, and traumatic brain injury, all of whom rely on the support of others for help accessing and making decisions about needed care. Older adults and individuals with disabilities accounted for 23% of Medicaid enrollment but 51% of spending,^9^ making them a particular group of interest.^10^

As is the case in other health care settings,^11^ many older adults and families receiving support from Medicaid HCBS are navigating challenges related to living with dementia. One‐third of Medicaid HCBS participants live with dementia.^12^, ^13^, ^14^ Some states provide additional dementia‐specific programs through HCBS waivers (e.g., dementia coaching).^15^ Home care agency staff report the importance of attending to specific needs of persons living with dementia, such as the significance of safety being noted in care plans, the need for additional communication with family members, and better understanding of older adult preferences for routine activities to reduce difficult dementia‐related behaviors.^16^ Therefore, the coordination of HCBS for older adults living with dementia is particularly complex and especially timely. For example, the Centers for Medicare and Medicaid Services (CMS) recognized the challenges in care coordination for this population, as evidenced by the recent implementation of the Guiding an Improved Dementia Experience (GUIDE) Model.^17^

### 1.1 Information Sharing in Medicaid HCBS

A critical yet challenging issue for Medicaid HCBS is the necessity of coordinating care and sharing information across various entities (e.g., state Department of Health, home care agencies, families) about participants' health, values, preferences, and treatments to ensure quality HCBS delivery.^18^, ^19^ Medicaid HCBS coordination often involves direct care workers (e.g., home health aides, personal care attendants) that provide hands‐on assistance with routine daily activities. Over the last several decades, states have increasingly allowed family caregivers to receive Medicaid payment to provide HCBS, thus placing them at the intersection of shared care networks. This model of care is particularly relevant for persons living with dementia, who may benefit from the familiarity of a family member providing care (versus a traditional home care aide).

Direct care workers manage a substantial amount of information about the health, function, and preferences for care of the individuals they serve.^16^, ^20^ Given the frequency of their presence in the home and the relationships they build with care recipients and their families, direct care workers are a necessary source of information when developing and refining plans of care. However, HCBS often lack systematic methods to enable care coordination and are complicated by circumstances like limited training and high turnover among direct care workers. Information sharing is often siloed and unstructured in HCBS, with this gap especially evident between direct care workers and care teams (e.g., nurses, care managers).^21^, ^22^, ^23^ Direct care workers report feeling unprepared when initiating services and being excluded from the Medicaid HCBS assessment and care planning process, making it difficult for them to contribute to and learn about participants' full scope of needs.^24^, ^25^, ^26^, ^27^ These issues are amplified when caring for persons living with complex conditions such as dementia and collaborating with family caregivers, who are also often excluded from structured information exchange.^28^ Systematic strategies to better leverage and efficiently integrate the expertise of direct care workers and caregivers in Medicaid HCBS care delivery are thus a critical aspect of improved care quality.^29^

### 1.2 Learning Health Systems (LHS) in Medicaid HCBS

LHS integrate technology, process, and policy to strengthen care delivery and quality that are products of efficient care coordination and information sharing.^30^ Existing literature largely focuses on LHS across medical services. We are unaware of LHS initiatives that are specific to Medicaid HCBS – but the Veterans Health Administration Veteran Directed Care (VDC) program is the closest comparison. Initiated in 2009 as the Veterans Directed‐HCBS program, the program follows a participant‐directed model, enabling veterans with functional or cognitive limitations requiring substantial assistance with daily activities to be allotted a monthly budget to purchase HCBS (e.g., personal care, home modifications).^31^ While its initial rollout was limited by budget considerations and personnel constraints,^32^ evidence demonstrates that the VDC is a valuable option for supporting veterans and their caregivers.^33^ Importantly, program evaluation involves assessment of health care utilization and costs with information from multiple sources, including the VHA's Corporate Data Warehouse (inpatient, outpatient, purchased care, cost data) and Medicare and Medicaid enrollment data. The VHA program coordinated across patient, worker, and system levels,^34^ each of which is critical to care delivery and quality in Medicaid HCBS, in which success is heavily reliant on how well people and components communicate, collaborate, and learn from each other.

Importantly, given its focus on person‐ and family‐centered care, transparency, and scientific integrity, LHS prioritizes health equity.^35^ HCBS participants encompass people from a range of experiences across race and ethnicity, sex, geography, language, and disability status.^36^, ^37^, ^38^ This diversity also extends to direct care workers.^12^, ^39^, ^40^ Inequities in care quality and access have been documented for participants, families, and direct care workers.^36^, ^41^, ^42^ Existing, fragmented systems collect information about participant health, function, and quality of life, as well as identify family caregivers and keep track of direct care workers' hours worked and tasks completed. Adopting LHS in Medicaid HCBS can help integrate current systems, as well as identify, evaluate, and address inequities in health and health care service use for participants from marginalized groups, those living in resource‐constrained areas, and those whose primary language is not English.^37^ Such information can enable states to understand whether programs are working to strengthen development efforts and allow for better resource planning for subpopulations when needed.

## 2 OBJECTIVE

**FIGURE 1:**
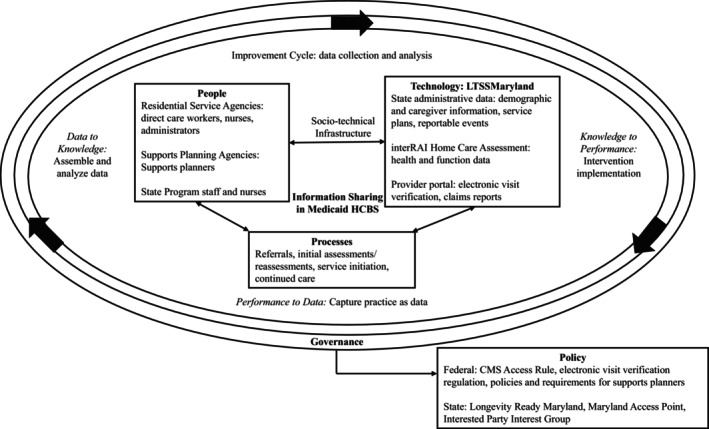
Information sharing in maryland medicaid HCBS learning health system.

This technical report connects the cyclical nature of processes to improve health care systems and applies an LHS' socio‐technical infrastructure, consisting of people, technology, policy, and processes to Medicaid HCBS.^43^ With Maryland as an example, we outline the components of a successful LHS with an emphasis on strategies to strengthen information sharing within Medicaid HCBS systems. Implementing an LHS framework in Medicaid HCBS requires translating methods and partnerships to new data sources and types of organizations, including state departments, home care agencies, and individuals, for direct care workers, family caregivers, and participants. We examine the utility of an LHS in Medicaid HCBS to strengthen information sharing in one state (Maryland) drawing on a framework presented by Friedman and colleagues (Figure 1)^43^ that depicts a successful LHS as requiring three linked components: (1) socio‐technical infrastructure, (2) improvement cycles, and (3) governance. The socio‐technical infrastructure consists of people, technology, policies, and processes that are necessary to the operation of improvement cycles that transition between performance, knowledge, and data. Improvement cycles refer to collecting and analyzing data to create and integrate knowledge into existing interventions to inform future iterations. Governance prioritizes communication to promote learning and improvement, and balances between “top‐down control and bottom‐up innovation.^43^” It should be noted that establishing and sustaining an LHS in Medicaid HCBS also requires buy‐in from a diverse group of stakeholders, adequate financial backing, purposeful design of data systems, partnership with academic or clinical institutions, and regulatory support.^44^

## 3 ENVISIONING A LHS IN MARYLAND HCBS

As is the case nationally, Maryland has experienced tremendous growth in Medicaid HCBS enrollment and expenditures. The number of Medicaid HCBS participants in Maryland increased by nearly 40% between 2013 and 2019.^45^ Between 2018 and 2020, HCBS expenditures in Maryland increased by nearly eight percent, from $2.1 billion to $2.3 billion.^3^ Maryland has rebalanced LTSS expenditures away from institutions and toward HCBS, with 62.4% of the state's LTSS expenditures being directed to HCBS in 2020.^3^ Maryland delivers HCBS to older adults and individuals with disabilities through a §1915(c) waiver (Community Options) and state plan (§1915[k] Community First Choice and Community Personal Assistance Services) options. Additionally, HCBS services are authorized through Maryland's §1115 demonstration waiver (Increased Community Services). All options require financial eligibility via income and/or asset limitations as well as functional criteria that indicate the level of need for services.^5^ Services range from those such as personal care, home‐delivered meals, assisted living, and medical day care. While an LHS has not been implemented in Maryland, existing systems that collect and maintain participant data have been used to better understand outcomes such as the risk of hospitalizations among dually eligible Medicare and Medicaid enrollees, as well as to develop methods to address delays in care resulting from being listed on Maryland's HCBS §1915(c) waiver registry for aging and disability services.^46^

### 3.1 Socio‐technical Infrastructure

Friedman and colleagues propose that LHS improvement cycles may be co‐occurring and are initiated by multistakeholder learning communities that conceptualize data collection from practice and move through discovery (i.e., data assembly, analysis, and interpretation) and implementation (i.e., intervention design and action).^43^ Essential to the success of improvement cycles is that LHS stakeholders embrace and acknowledge uncertainties that may exist along the way, and that socio‐technical infrastructures are created to support multiple co‐occurring improvement cycles.^47^ Additionally, an effective LHS requires governance to ensure ethical implementation of an LHS through oversight of stakeholders, advocates, and families.

The socio‐technical infrastructure is described as a set of integrated services (e.g., learning communities, tools to measure performance, delivery of interventions) that exist across people, technologies, processes, and policies necessary for generating the data required for successful improvement cycles.^43^ Components of the socio‐technical infrastructure and likely improvement cycles and governing processes in the context of Medicaid HCBS in Maryland are described below. Table 1 organizes Medicaid HCBS delivery as a matrix, highlighting delivery components (referral to HCBS, assessment, service initiation, care continuity), entities involved in each component (i.e., people), their responsibilities (i.e., processes), and the technologies used throughout the HCBS care delivery process. As HCBS policy provides guidance for the tools, systems, and processes that are needed to support the day‐to‐day operations of care delivery, one key difference in our LHS structure is that policies are organized as part of the governing process, rather than as part of the socio‐technical infrastructure.

**TABLE 1:** Maryland medicaid HCBS and LTSS Maryland across HCBS implementation stages.

Implementation stages	People (Entity)	Participates	Process (Responsibilities)	Technology (LTSS *Maryland*)			
				Demographics	Service plan	InterRAI	EVV
1. Referral	State	X	Receives referral for HCBS via Maryland Access Point; nurse assessor completes InterRAI HC assessment with participant and family to determine eligibility	X		X	
	Supports planner	No action at this stage					
	RSA						
	Direct care worker						
2. Supports planning	State	X	Review/approve plan of service	X	X	X	
	Supports planner	X	Conducts person‐centered planning with participant; submit proposed service plan to Department of Health	X	X	X	
	RSA	No action at this stage					
	Direct care worker						
3. Service initiation	State	X	Monitors initiation of services		X		X
	Supports planner	X	Collaborates with participant and family to notify RSA of plan approval		X		
	RSA	X	Receive referral for services; signs plan of service; assign direct care worker; initiate services; oversight of services		X		
	Direct care worker	X	Initiate services with participant (and family representative if appropriate)				X
4. Care continuity	State	X	Review service plan changes (submitted by supports planner) as needed		X	X	
	Supports planner	X	Conduct monthly phone check‐ins; quarterly in‐person visits, annual assessments; revises service plan		X	X	
	RSA	X	Monitor participant needs; supervise direct care workers				X
	Direct care worker	X	Provide services to participant; collaborate with family				X
**All Stages**	HCBS participants/personal representatives may access their record or engage in supports planning in LTSS*Maryland*.						

Abbreviations: EVV, Electronic Visit Verification; HCBS, Home and Community‐Based Services; interRAI HC, interRAI Home Care; RSA, Residential Service Agency.

While we describe the traditional trajectory of HCBS delivery, it is important to note considerations related to care coordination and information sharing for persons living with dementia. For example, coordination of assessments, service initiation, and ongoing care often falls to family caregivers.^48^ HCBS program constraints that prolong the time between initial referral and initiation of services may result in financial, physical, and emotional implications for family caregivers. The additional demands placed on dementia family caregivers in Medicaid HCBS warrant attention and innovative supports that an LHS in Medicaid HCBS has the potential to address.^49^ Our prior work in Maryland describes dementia caregivers as more involved in their family member's home care services, relative to non‐dementia caregivers.^16^ Dementia family caregivers also more often experience emotional burden that may result from additional responsibilities, like managing direct care workers in the home.^41^, ^48^

#### 3.1.1 People

Friedman and colleagues define “people” as members of the trained workforce. Due to the longitudinal and noninstitutional setting of HCBS, persons involved in Medicaid HCBS delivery are situated across several organizations. The state department of health is responsible for the oversight of the Medicaid HCBS program. Its staff include nurses who are responsible for assessing potential participants for eligibility, as well as medical care specialists who review and approve care plans. Care management (“supports planning” in Maryland) services are delivered through local Area Agencies on Aging and private agencies that are competitively solicited and hold contracts with the state department of health. Supports planners are responsible for coordinating home care services which involve standard periodic in‐person visits, as well as monthly check‐in calls. They also act as liaisons between state programming staff, care recipients and their families, and service providers. Home care agencies in Maryland (referred to as “residential service agencies”) provide personal care services alongside home health services.^50^ Residential service agency staff (e.g., administrators, nurses) assign direct care workers to provide services and maintain oversight of day‐to‐day home care delivery. Notably, unlike some states, Maryland does not require clinicians (e.g., primary care providers) to approve care plans for Medicaid HCBS. In states where approval is required, the role of clinicians should be considered in the development of a LHS, as they are sources of accurate and up‐to‐date health information for participants. As LHS have been largely studied in medical settings, one in HCBS could leverage similar principles for integrating information and expertise from clinicians.

For older adults living with dementia, additional coordination efforts take place between all members of the care team and families to ensure proper care coordination and service delivery.^12^, ^16^ First, direct care workers are equipped with varying levels of relevant training, although some training gaps may be addressed in the coming years.^12^ Recent state legislation requires that residential service agencies provide dementia training to home care aides.^51^ Second, direct care workers may include family members, since Maryland allows family members to be paid to deliver HCBS, as is the case in most other states.^52^

Despite family caregivers being able to be paid to care for HCBS participants, most are unpaid. While not often considered part of the trained workforce, they are a primary source of information and support. Their observations are critical during, as well as between, HCBS reassessments and ongoing care delivery. Family caregivers interact longitudinally with home care aides and providers and are involved in most aspects of care delivery, but there are minimal systematic processes for integrating them into current HCBS policies and practices.^48^, ^53^ In Maryland, one family member of an HCBS participant is their documented representative. The representative acts as the emergency or primary contact. Little information is obtained about family representatives outside of case notes (i.e., free text) and home care assessments, including a formal understanding of the type and amount of help they will provide to HCBS participants. An LHS would include information about family caregiver roles and contributions to provide a complete picture of the type of assistance participants receive. Such information could be collected during initial assessments and updated annually or at key junctures (e.g., hospital discharge).

#### 3.1.2 Technology

Technologies refer to modalities that support people in carrying out the work of improvement.^43^ Although the LHS is largely based on the integration of systems such as patient electronic health records (EHR) and no state Medicaid program (including Medicaid HCBS) has yet employed an EHR system,^54^ other rich data sources are available and heavily used (e.g., functional assessments, electronic visit verification [EVV]). Additionally, in contrast to systems like the patient portal (online platforms that enable patients to view health information, perform health management tasks, and interact with clinicians),^55^ HCBS technologies are lower cost than those in institutional settings and are often geared toward information management. In Maryland, members of the HCBS care team coordinate care delivery through an electronic management system called LTSS*Maryland*. LTSS*Maryland* was established to facilitate the coordination, delivery, quality oversight, and payment for Medicaid and state‐funded LTSS programs in Maryland.^46^ LTSS*Maryland* is similar to systems used by other states^56^, ^57^ and holds HCBS participant sociodemographic and legal information, service plans, reportable events, and data collected as part of the interRAI Home Care assessment, which collects information on a regular basis to reflect changes in health and function, and document emergency room visits, hospitalizations, and care circumstances to help inform care planning.^58^, ^59^ A recent initiative that has expanded technologies in Maryland to better understand the experience of HCBS participants is the linkage of the Chesapeake Regional Information System for our Patients (CRISP) to LTSS*Maryland*. CRISP is a state‐designed health information exchange that provides information about hospital admission, discharge, and transfers.^60^ This linkage opens the possibilities of including information from clinicians in an LHS for HCBS. Access to participant data is driven by care team members. However, not all data is accessible to all care team members, which may result in information gaps between direct care workers, residential service agencies, and state providers (Table 2).^58^, ^59^

**TABLE 2:** Function and Accessibility in LTSSMaryland.

Component of LTSSMaryland	Function/ Purpose	Entity/Individual accessibility				
		State	Supports planner	RSA	Direct Care Worker	Individual and/or Family*
Participant demographic information	Collects participant contact and demographic (e.g., age, race/ethnicity, sex), family representative (i.e., primary family caregiver), and legal information (e.g., conservatorship, power of attorney).	X	X			X
Service Plan	Documents participant goals, approved services to include frequency, units, and provider, identifies emergency plan, unpaid supports (e.g., family support), other Medicaid services. Captures signatures for all approved providers. Documents health and safety needs are met with established plan.	X	X	X		X
interRAI Homecare Assessment	Collects information about participants' function, social factors, caregiver burden, and health care utilization on an annual basis	X	X			X
Electronic Visit Verification	Collects information about location of service delivery, care activities, and arrival and departure times of direct care workers. Generates claims for services.	X	X	X	X	X

*Note*: *HCBS participants/personal representatives may access their record or engage in supports planning in LTSS Maryland.

As is the case in other states, Maryland collects information about participants and families in several ways.^56^ For example, HCBS providers in Maryland rely on the interRAI Home Care Assessment. Other routinely collected data include Medicare and Medicaid administrative claims and EVV, which collects information about service delivery, care activities, and arrival and departure times of direct care workers.^61^ While EVV is typically used to assess resources and combat fraud and waste in HCBS, it could serve as a tool to assess and address impacts of workforce turnover and overtime on HCBS care quality. Maryland leverages LTSS*Maryland* to hold pertinent information about program interest (registry/waitlists), referrals to HCBS, financial eligibility determinations, functional assessments to determine level of care, service plan development and approval, and the ongoing monitoring and delivery of services. Residential service agency administrators and direct care workers have access to a provider portal that includes EVV, claims reporting, and reportable events documentation. However, they do not have access to information captured in the interRAI Home Care Assessment, which would enable a streamlined communication process and improve their understanding of care recipients' and family caregivers' needs and experiences.

#### 3.1.3 Processes

Processes refer to routines that enable work to be carried out efficiently.^43^ In Maryland Medicaid HCBS, several actions take place along the continuum of service delivery. The multistep process involved in HCBS service delivery and information exchange is described below (also depicted in Table 1).

1. Referral: Individuals are referred to HCBS via Maryland Access Point, which was established in response to Medicaid's No Wrong Door policy to increase access to LTSS.^62^ Referrals are accepted from all sources. Nurse assessors complete interRAI Home Care Assessments in LTSS*Maryland* to determine medical eligibility for HCBS. LTSS*Maryland* generates level of care (LOC) determinations based on interRAI Home Care submissions and the state's LOC criteria with oversight from the utilization control agent.
2. Supports planning: Once the level of care determination is available in LTSS*Maryland* and the participant is medically eligible for HCBS, a supports planner coordinates a person‐centered planning meeting. This process often includes family caregivers to assist with developing a plan of service that highlights participants' goals, selects services, identifies providers, and ensures health and safety. The plan of service is submitted to the Department of Health for approval.
3. Service initiation: Following service approval, supports planners work with participants and families to initiate and continue services with providers. HCBS may be provided by home‐delivered meal organizations, medical day care services, by direct care workers via residential service agencies, etc.
4. Care continuity: Supports planners conduct monthly phone check‐ins, quarterly face‐to‐face visits, and assist with the annual redetermination process. Ongoing care includes ensuring participants' needs are being met with the supports outlined in the plan of service, addressing issues related to service delivery (e.g., challenges with the direct care worker), pausing and restarting services (e.g., hospitalizations), requesting assessments to document changes in functional status, and submitting service plan change requests to the Department of Health.

#### 3.1.4 Improvement cycles

Improvement cycles refer to the ongoing processes that LHS use to collect and analyze data to generate knowledge that informs intervention development that yields new data. Improvement cycles consist of services that are essential to data collection and analyses to strengthen the LHS, including: performance to data (captures practice as data), data to knowledge (the process of assembling and analyzing data), and knowledge to performance (intervention implementation). In Maryland, one example of an improvement cycle that exists organically is the partnership between the Maryland Department of Health and The Hilltop Institute, a nonpartisan research organization at the University of Maryland Baltimore County. The Hilltop Institute engages in projects with states across the country. In Maryland, Hilltop maintains Maryland Medicare and Medicaid data, hospital discharge data, nursing home assessment data, data on commercially insured individuals, and interRAI Home Care assessments for Medicaid HCBS participants. The Hilltop Institute makes Medicaid and HCBS enrollment data publicly available through monthly updates to their Maryland Medicaid DataPort. They also conduct studies that improve care delivery in Maryland Medicaid HCBS. For example, in efforts to address Maryland's Medicaid Community Options Waiver registry, Hilltop developed an algorithm to predict an individual's risk of long‐term institutionalization based on demographics, clinical acuity, functional status, and health services utilization. The Maryland Department of Health implemented Hilltop's model to target 80% of waiver openings for care recipients at the greatest risk.^45^, ^63^

### 3.2 Governance

A successful LHS should “strike a balance of top‐down control and bottom‐up innovation”.^43^ Recommendations to ensure governance in an LHS include: (1) the implementation of a governance board representing a variety of stakeholders, including HCBS participants, family caregivers, and providers, (2) transparency in LHS goals and outcomes between providers, HCBS participants, and families, and (3) trust that data is being collected, stored and shared ethically.^64^, ^65^ Such efforts would enable diverse groups of stakeholders to contribute to quality assurance and improvement processes by which compliance standards can be developed and enforced, and data‐driven analysis can improve HCBS processes.^66^ Feedback and accountability administered via governance efforts in an LHS in Maryland Medicaid HCBS may include the already established Community Options Advisory Council, which exists to enable discussion about policy, planning, and issues with stakeholders, including advocates, providers and participants. Additionally, in response to the CMS Medicaid Access Rule calling for states to establish Beneficiary Advisory Councils comprised of Medicaid beneficiaries, families, and/or caregivers, Maryland will establish the Interested Parties Advisory Group in 2026, which will create a structured platform for collaboration among stakeholders including direct care workers, employers, consumer advocacy groups, and other relevant organizations. The Advisory group will also monitor processes and annual reports to ensure that the needs of those directly impacted by the LHS are met.^67^ Collaboration between the advisory group and the Department of Health provides an opportunity for HCBS stakeholders to contribute to the oversight and monitoring of the LHS.

#### 3.2.1 Policy

Policies shape how work is performed and can cross federal and state levels. This may include strategies related to the allocation of HCBS expenditures, reduction in the state's waiver registries, and investments in workforce support. For example, in April 2024, the CMS passed the Medicaid Access Rule. In addition to increasing direct care worker wages and improving equitable access to HCBS, the final rule requires that states report on nationally standardized quality measures for HCBS established by CMS, which will be developed through a stakeholder process and public comment.^68^ To advance equity in HCBS, states will be required to report quality measures stratified by factors likerace, ethnicity, sex, age, rural/urban status, disability, and language. While measure development and implementation processes are not yet clear, it is feasible that data from LTSS*Maryland* could be leveraged to measure care quality. Additionally, Medicaid HCBS programs follow regulations related to the 21st Century Cures Act, which mandates that states implement EVV for all Medicaid personal care services and home health services^69^

Because states administer and oversee Medicaid HCBS programs, efforts to better support participants will vary. In January 2024, Governor Wes Moore issued an executive order establishing *Longevity Ready Maryland*, a multisector initiative to prepare the state for its growing aging population.^70^, ^71^ *Longevity Ready Maryland* is guided by four goals: (1) build a longevity ecosystem, (2) promote economic opportunity, (3) prepare Marylanders to afford longevity, and (4) optimize health, wellness, and mobility across priorities that include caregiving, the care workforce, supportive communities, and affordable housing. The initiative will coordinate services to address the economic and social needs that help support the aging population in Maryland. Policy recommendations that emerge from *Longevity Ready Maryland* will likely impact Medicaid HCBS accessibility and the support of its workforce. Similarly, Maryland developed a 5‐year (2022–2026) state plan to address dementia. Drafted by the Virginia I. Jones Alzheimer's Disease and Related Disorders Council, the plan names goals to improve dementia care in the state, including enhanced quality, access, and coordination of ADRD care; expanding supports for family caregivers; advancing dementia research; and improving data capabilities related to dementia and its impact, and the effects of interventions. A successful LHS would leverage discoveries from improvement cycles that result from the implementation of policies and analyze them to better inform next steps.

## 4 CONCLUSION

The provision of HCBS is complex and varies by state but requires the involvement of a wide range of entities that include state agencies, home care providers, direct care workers, and families. The coordination of care and information sharing between state departments, home care agencies, and families about participants' health, values, preferences, and treatments is an especially challenging issue that may adversely affect HCBS care quality. This technical report proposes the implementation of LHS in Medicaid HCBS, using Maryland HCBS as an example to highlight the value in states deliberatively integrating and using data to improve HCBS care quality. An LHS in Medicaid HCBS would reinforce health equity and strengthen states' public health planning by enabling them to improve HCBS feedback and accountability, data systems linking, and interoperability and harmonization across multiple programmatic efforts.

We describe the value of LHS approaches that build on the availability of data and technologies, such as electronic management systems (i.e., LTSS*Maryland*) and care coordination efforts required across and through multiple care team members (people) and processes to ensure optimal care delivery in Maryland. The governance of an LHS in HCBS calls for engagement from shareholders including HCBS participants, families, and providers, a strategy that is part of recent CMS policy changes to strengthen HCBS.^68^ States implementing LHS in their HCBS programs would benefit from bidirectional relationships between health systems and researchers using data and evidence to improve care delivery using equitable and innovative methods.^72^ Strategies are needed to pilot and implement an LHS in Medicaid HCBS that stands to improve outcomes, efficiency, and equity.

## FUNDING INFORMATION

Funds to support this work were provided by the National Institute on Aging (K01AG080079) to C. D. F.

## CONFLICT OF INTEREST STATEMENT

The authors declare no conflicts of interest.

## Data Availability

Data sharing is not applicable to this article as no new data were created or analyzed in this study.
